# Exploring Texture and Biomechanics of Food Oral Processing in Fork-Mashable Dishes for Patients with Mastication or Swallowing Impairments

**DOI:** 10.3390/foods13121807

**Published:** 2024-06-08

**Authors:** Kovan Ismael-Mohammed, Mireia Bolívar-Prados, Laura Laguna, Adrian Nuñez Lara, Pere Clavé

**Affiliations:** 1 Gastrointestinal Physiology Laboratory, Hospital de Mataró, Universitat Autònoma de Barcelona, 08304 Mataró, Spain; 2 Institute of Agrochemistry and Food Technology (IATA, CISC), 46980 Valencia, Spain; 3 Centro de Investigación Biomédica en Red de Enfermedades Hepáticas y Digestivas (Ciberehd), 08304 Barcelona, Spain

**Keywords:** dysphagia, textural properties, Texture E BDA/IDDSI level 6, fork-mashable

## Abstract

Texture-modified diets (TMDs) are a primary compensatory treatment for hospitalized older patients with swallowing and mastication disorders. Nevertheless, the lack of a protocol for evaluating their objective textural properties hampers their industrialization and optimal patient care. Objectives: This study aimed (a) to evaluate the textural properties (maximum force, cohesiveness, and adhesiveness) and biomechanics of food oral processing (mastication cycles, time, and frequency) of ten fork-mashable dishes (Texture E BDA/IDDSI level 6), (b) to explore the impact of oral processing on texture, and (c) to measure the properties of the ready-to-swallow bolus (RSB) in healthy adults. Methods: The textural properties (maximum force, cohesiveness, and adhesiveness) of ten dishes were analyzed with a texture analyzer before and after oral processing (RSB) in five healthy adults (30 ± 3.9, 3 women). Surface electromyography was used to measure mastication cycles, time, and frequency. Results: The pre-mastication Texture Profile Analysis (TPA)-averaged values of maximum force ranged from 0.65 to 2.73 N, cohesiveness was 0.49–0.87, and adhesiveness was 0.01–0.95 N·s. Masticatory Cycles (46.87–19.13 MC) and time (36.73–15.80 S) from whole samples to RSB greatly and significantly differed among dishes, although frequency did not (1.68–1.11 MC/T). Post-mastication RSB TPA-averaged values of maximum force ranged from 0.70 to 2.24 N; cohesiveness, 0.49–0.73; and adhesiveness, 0.01–1.14 N·s. Conclusions: Despite all dishes being classified by the same qualitative descriptor (BDA level E/IDDSI level 6), there was a large and significant variation in their textural properties (maximum force, cohesiveness, and adhesiveness) when measured in SI units. In addition, in healthy adults, the masticatory cycles and time to achieve RSB greatly differed, whereas masticatory frequency remained quite constant.

## 1. Introduction

Aging causes transformations which affect older people’s physical and cognitive capacities [1,2]. Getting older significantly affects the processes of mastication and swallowing [3,4] and may lead to several impairments, such as oropharyngeal dysphagia (OD) [5,6]. These difficulties impact health and may lead to inadequate nutrition, dehydration, and aspiration pneumonia, ultimately resulting in a diminished quality of life [7,8].

A large part of the aging population suffers from mastication impairments. A study by Dias-da-Costa’s et al. (2010) in Brazil found that 49.7% of participants reported poor mastication [9], and Murrieta et al. (2016) in Mexico discovered a 33.1% prevalence of temporomandibular disorders among 60–69-year-old patients [10]. Dibello et al. (2021) identified reduced oral motor skills, including masticatory function, as contributing factors to frailty in older adults [11]. Furthermore, swallowing disorders among older adults range from 10% to 33% [12]. Remarkably, research found that the prevalence of dysphagia among older individuals living independently was 16.6% for those aged between 70 and 79, rising to 33% for individuals over 80, and reaching 47% among hospitalized older individuals [13]. 

The Test of Masticating and Swallowing Solids (TOMASS) was created to address the clinical need to evaluate mastication and swallowing disorders. It was specifically developed for studying swallowing impairments in individuals with Parkinson’s disease [14] and modeled on the Timed Water Swallowing Test (TWST), but substituting a solid bolus texture (usually cracker) instead of liquids. The data collected from this test that are considered to be important are the number of bites, the number of masticatory cycles, and multiple swallows [15]. The TOMASS focuses on oral bolus preparation, addressing an important aspect that is often overlooked in current assessments and provides objective insights into the efficiency and speed of mastication. In addition, surface electromyography (EMG) measures extracted from the masseter muscles were closely correlated with observations of mastication cycles, and they add reliability and validity to the TOMASS data [16]. 

Another limitation to research in this field is the current lack of standardized terminology for TMDs, leading to wide variation in the degree of texture modification in their descriptions. This lack of standardization can create confusion and inconsistencies in dysphagia management across countries and healthcare settings. By establishing international standards based on measurements in International System of units (SI) of the rheological and textural properties causing the therapeutic effect of these diets, healthcare professionals can ensure a consistent and universal understanding of texture-modified diets, improving reproducibility and quality control and, thus, leading to improved patient safety and treatment outcomes [17].

There have been several attempts to solve this problem, and healthcare institutions have developed TMDs for patients based on several classifications, such as the International Dysphagia Diet Standardization Initiative (IDDSI), the British Dietetic Association (BDA), and the Smile Care System or the triple adaptation of the Mediterranean diet, all of which provide qualitative characterization. Currently, there is no standardized protocol for determining optimal texture in TMDs for patients with mastication and swallowing disorders. IDDSI offers an eight-level classification for liquid and solid foods, using qualitative descriptors and empirical methods [18]. The British Dietetic Association (BDA) developed their own subjective descriptors (B—thin puree; C—thick puree; D—pre-mashed diet; and E—fork-mashable diet) [19]. The Smile Care System categorizes texture-modified foods based on mastication and swallowing ability—blue for healthy, yellow for mastication impairments, and red for swallowing impairment [20]. The Triple Adaptation of the Mediterranean diet aligns 296 dishes with the BDA Texture C (thick puree) and Texture E (fork-mashable) [21]. In our hospital, Texture-E dishes are prepared for patients with mastication impairments, and Texture-C dishes for patients with swallowing impairments. Recent clinical trials evaluating the therapeutic effect of these diets on older patients with oropharyngeal dysphagia studied the process of oral processing—breaking down and mixing food with saliva before swallowing [22].

To replicate oral food processing, Friedman, Whitney, and Szczesniak introduced the Texture Profile Analysis (TPA) in 1963, using a texture analyzer and identifying textural characteristics from the initial two bites. It is popular with the food industry for its simplicity and ability to assess textures similar to those produced during oral processing [23]. In vitro studies simulate mechanical and enzymatic processes, while in vivo studies observe muscular activity and organ function during food consumption. Previous studies focused on solid foods requiring more chewing, such as meat, fruits, vegetables, bread, and cereal. We recently developed a rheological protocol to replicate oral processing and pharyngeal factors affecting the therapeutic effects of alimentary fluids, reporting viscosity measurements in a standardized manner. 

In this study, we aimed to assess the textural properties according to TPA (maximum force, N; cohesiveness; and adhesiveness, N·s) of ten fork-mashable (BDA Texture E and IDDSI level 6) dishes in a hospital setting, specifically designed for patients with swallowing and mastication disorders, and to understand the effect of mastication and oral processing on the properties of the ready-to-swallow bolus (RSB) and biomechanics of food oral processing (mastication cycles, time, and frequency). 

## 2. Materials and Methods

### 2.1. Materials 

#### 2.1.1. Texture-Modified Diets 

A selection of 10 TMDs from the triple adaptation of the Mediterranean diet for older people with oropharyngeal dysphagia [21] was provided by Catering Arcasa SL (Esplugues de Llobregat, Spain) and elaborated at Mataró Hospital. These dishes were classified as fork-mashable BDA Texture E or IDDSI level 6 classification system (Table 1 and Table 2). The specific dishes included in the study were (1) French omelet, (2) zucchini omelet, (3) pumpkin, (4) stewed turkey, (5) pollock fish, (6) red Lentils, (7) noodles, (8) hake; (9) cauliflower, and (10) broccoli.

**Table 1:** Characteristics and texture check for the fork-mashable (Texture E) according to BDA qualitative classification [19].

	Texture Check	Texture Characteristics
Foods	Mashable with fork	Soft, tender, moist.
Thick fluids	Sauce, custard (spoon stands)	Does not require chewing
Meat	Soft, no bigger than 15mm, minced, in thick sauce	Mashable with a fork
Fish	Soft, breakable with a fork	No mixed textures
Fruit	Mashed, drained juice	No thin fluids
Casseroles/stews/curry	Thick, prepared meat/fish/veg., mixed	No hard, tough, chewy, fibrous, dry, crispy bits
Cereal	The texture of thick smooth porridge or fully softened cereal	No pips, seeds, or pith/inside skin
Desserts	The texture of thick smooth yogurt, stewed apple in custard, or soft cake with thick custard	No round/long foods, hard chunks, sticky foods, “floppy” foods. No juicy food with separating juice or hard pieces from cooking, and no thinned or separated fluids

**Table 2:** Texture check and characteristics for level 6 (soft, bite-sized) according to IDDSI qualitative classification [24].

	Texture Check	Texture Characteristics
Meat	Tender and chopped into 1.5 cm × 1.5 cm pieces; serve minced and moist if not soft	Soft, tender and moist, with no thin liquid
Fish	Soft-cooked, served in 1.5 cm × 1.5 cm pieces	Mastication is required
Fruit	Soft, chopped into 1.5 cm × 1.5 cm pieces, drained; avoid fibrous parts and be cautious with high water content	Bite-sized pieces, so they are safe to swallow
Vegetables	Steamed or boiled, final size 1.5 cm × 1.5 cm; no stir-frying	The bite-sized pieces no bigger than 1.5 cm × 1.5 cm in size
Cereal	Pieces no bigger than 1.5 cm × 1.5 cm, fully softened, excess liquid drained	Mashable with fork
Rice	Moistened with a non-pouring sauce, not sticky; individual grains should not separate	No knife is required to cut off the food

#### 2.1.2. Texture-Modified Diets Preparation 

The TMDs under investigation were prepared in a hospital kitchen according to the Dysphagia Diet Food Texture Descriptors of the BDA standards for Texture E (fork-mashable). All the dishes were tested qualitatively for both BDA and the IDDSI classifications in order to make sure that they fulfilled the descriptions of both.

Table 3 provides an estimation of the fork-mashable compositions, information on ingredients and proportions in percentages, including carbohydrates, proteins, and kilocalories, for all dishes tested. Pictures of the selected dishes and their cooking method are shown in the Supplementary Materials, showing the dishes in a readily fork-mashable state. 

#### 2.1.3. Participants

The study included five healthy adults, three women and two men, average age 30 ± 3.9 year, with no reported health issues. Selection criteria consisted of individuals aged between 18 and 40, capable of effective chewing, with a normal dental status, and willing to give informed consent. Exclusion criteria consisted of the failure to meet these specifications, an inability to finish all 10 dishes, or the presence of conditions like Sjögren’s syndrome or excessive saliva production. Healthy adults were chosen for their normal chewing skills and sufficient saliva production necessary for creating an optimal bolus during the oral phase.

#### 2.1.4. Study Design 

This study aimed to analyze the textural properties of 10 dishes during both the pre- and post-stages of oral processing. Five healthy adults participated in the study, which was approved by the Ethics Committee of the Consorci Sanitari del Maresme (Code 63/22). Participants were instructed to process 30 g in triplicates of each dish, 3 × 30 g, and to spit out the bolus when ready to swallow. The bolus was analyzed to evaluate its textural properties, while EMG recordings were taken during oral processing to measure the activity of the masseter and temporalis muscles involved in chewing. This approach provided for a comprehensive understanding of the oral-processing biomechanics.

### 2.2. Method 

Three separate batches of each BDA Texture E (IDDSI level 6) dishes were selected for textural analysis, each batch was transported individually to the lab. The dishes were analyzed at 40–50 °C at pre-mastication [23], the standard serving temperature for patients with dysphagia and chewing problems.

#### 2.2.1. Texture Profile Analysis 

**Figure 1:**
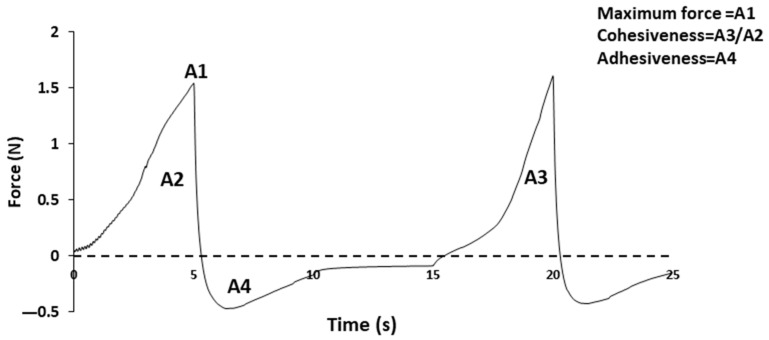
The Texture Profile Analysis (TPA) and the parameters obtained: maximum force (N), cohesion, and adhesiveness (N·s).

The TPA, as shown in Figure 1, based on Bourne et al. (1978), was performed on the 10 dishes of Texture E, using the TA. XT Plus Texture Analyzer from Stable Micro Systems (Stable Micro Systems, Godalming, UK). The tests were repeated 15 times for pre-oral and 15 times for post-oral processing. The texture analyzer cell was loaded with 5 kg of force and a 0.049 N trigger force, using an aluminum cylinder probe with a diameter of 36 mm paired with an acrylic recipient. The TPA test was performed by introducing 30 g of the dish into the recipient at 40–50 °C, with a test speed of 1 mm/s. The results showed maximum force, cohesiveness, and adhesiveness for each dish in SI units. Data collection and management were performed using Stable Microsystems’s exponent software (version 7.0.6.0). The first peak of the TPA graphs was taken as the maximum force, the first negative area was counted as the adhesiveness, and the cohesiveness equaled the second positive area divided by the first positive area [25].

#### 2.2.2. Assessment of Mastication by Electromyography (EMG)

**Figure 2:**
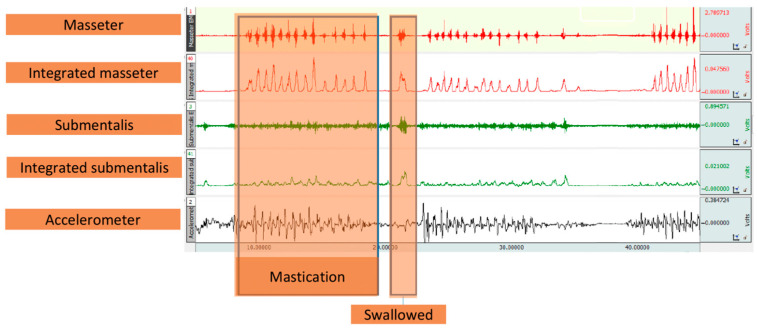
The signals generated by the effect of food mastication during the TOMASS.

We adapted the Test of Masticating and Swallowing Solids (TOMASS) as a quantitative assessment of oral processing [16]. Participants were seated, and electrodes were placed on their masseter and sub-mentalis muscles and a bone structure (right wrist and behind the right ear) for the ground, using alcohol to prepare the skin. An accelerometer was placed under the Adam’s apple, and a Logitech C920 PRO HD Webcam recorded the test for visual analysis. Healthy adults were instructed to eat a food sample as quickly as possible and raise their hands when ready to swallow. The video was analyzed for masticatory cycles and total time to swallow using the Acknowledge 5.0 program from Biopac Systems, Inc. EMG registers measured the amplitude, frequency, and duration of each masticatory cycle, as shown in Figure 2. 

#### 2.2.3. Ready-to-Swallow Bolus

The TPA was performed on all RSB spat out after mastication, using the method explained in Section 2.2.1. 

#### 2.2.4. Assessment of the Effect of Oral Mastication—Impact Percentage 

There were 3 types of parameters: (a) absolute differences in each parameter (pre–post), (b) relative effect of mastication (oral mastication impact percentage), and (c) relative effect on pre-mastication textural parameters. The absolute differences in each parameter between the pre-mastication and post-mastication were calculated according to the following formula: highest value–lowest value. The effects on textural characteristics (maximum force, cohesiveness, and adhesiveness) were calculated and expressed in SI units. The highest values obtained during the measurement of pre- and post-oral mastication were used as reference values to measure the effect with the texture analyzer, using the following formula: [(highest values − lowest value/highest value)] × 100 [25]. For the relative effect on the pre-mastication, the calculation was performed using the following formula: (absolute value/pre-mastication value) × 100%.

### 2.3. Data Management and Statistical Analysis

Each fork-mashable dish underwent three separate analyses per day over five days. A one-way ANOVA analysis examined the variation in textural properties among dishes before and after mastication. Similarly, a one-way ANOVA was applied to assess differences in the assessment of mastication across all dishes. To identify the least significant differences, the Tukey test was performed, with a significance level set at *p* < 0.05. All statistical analyses were performed using XLSTAT 2020.4.1 (Addinsoft, Paris, France). 

To address values exceeding 100%, we employed a simple adjustment method. (a) Assessment of exceeding values: For the relative effect on pre-mastication textural parameters, we identified values in the dataset that exceeded 100%. (b) Calculation of total sum: The total sum of these exceeding values was computed. (c) Determination of adjustment factor: An adjustment factor was computed by dividing 100 by the total sum of the exceeding values. This factor indicates how much each value needs to be scaled down to ensure that the total sum does not exceed 100%. Or (d) adjustment of exceeding values: Using the adjustment factor, each exceeding value was scaled down proportionally. This ensured that the sum of all adjusted values remained close to 100%.

## 3. Results

**Figure 3:**
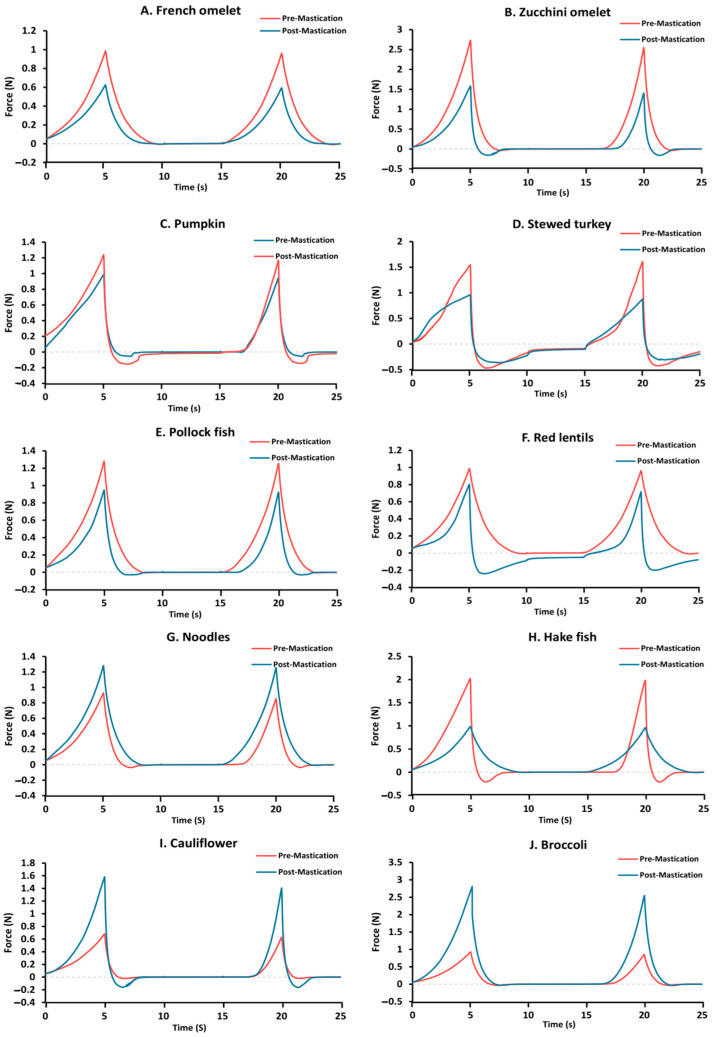
Examples of the curves obtained with the TPA test of the 10 fork-mashable pre- (red tracing) and post- (blue) oral processing: (**A**) French omelet, (**B**) zucchini omelet, (**C**) pumpkin, (**D**) stewed turkey, (**E**) pollock fish, (**F**) red lentils, (**G**) noodles, (**H**) hake, (**I**) cauliflower, and (**J**) Broccoli.

All 10 dishes fulfilled the descriptions of BDA for fork-mashable dishes (Texture E) and also for IDDSI level 6. TPA curves for the 10 TMDs before and after oral mastication are shown in Figure 3. The data are presented in Table 4, Table 5 and Table 6, along with the percentage of the oral mastication effect, absolute differences, and relative effect on the pre-mastication. 

### 3.1. Mechanical Characteristics 

**Figure 4:**
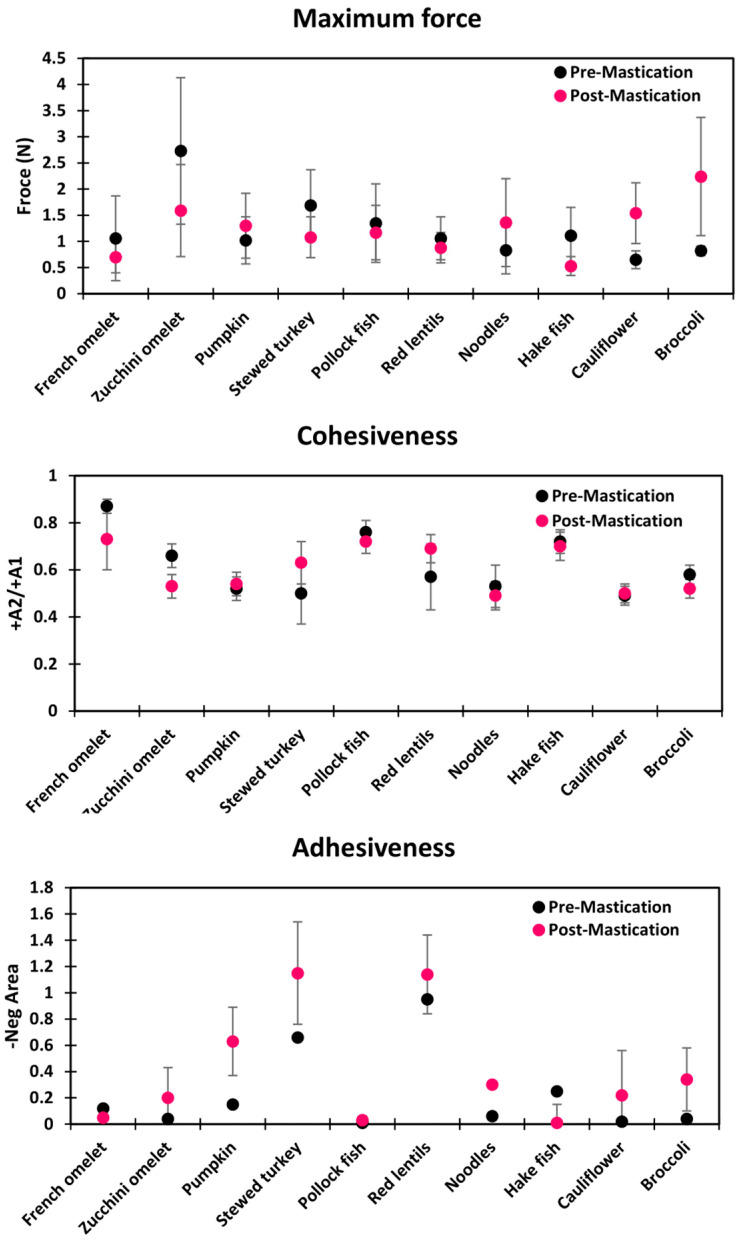
Mean values for maximum force, cohesiveness, and adhesiveness for all 10 fork-mashable dishes.

The maximum force (N), cohesiveness, and adhesiveness results at pre- and post-oral processing for each TMD and the effect of oral mastication on the textural characteristics are presented in Table 4, Table 5 and Table 6 and Figure 4.

#### 3.1.1. Pre-Mastication

The zucchini omelet showed the highest force (2.73 ± 1.40 N) among all the dishes tested, while cauliflower, at 0.65 ± 0.17 N, displayed the softest texture (76.19% variability). Cohesiveness varied by 40.22% among dishes. The French omelet had the highest cohesiveness (0.87 ± 0.03), while pumpkin had the lowest (0.52 ± 0.05). Furthermore, adhesiveness showed diverse results: red lentils ranked highest in adhesiveness (0.95 ± 0.47 N·s), whereas pollock fish exhibited the least (0.01 ± 0.01 N·s) (98.94% variability).

#### 3.1.2. Post-Mastication/Ready-to-Swallow Bolus

Following oral processing, the zucchini omelet RSB presented the highest maximum force (1.59 ± 0.88 N), and hake fish exhibited the least maximum force (0.53 ± 0.18 N), (66.66% variability). Concerning cohesiveness, the French omelet had the highest value at 0.73± 0.13, while noodles had the lowest (0.49 ± 0.06) (32.37% variability). In terms of adhesiveness, significant differences among the foods were evident. Stewed turkey showed the highest adhesivity, with a value of 1.15 ± 0.39 N·s, and hake fish displayed the least stickiness, with an adhesiveness value of 0.01 ± 0.01 N·s (99% variability).

#### 3.1.3. Mastication and Oral-Processing Effect 

For maximum force, the zucchini omelet decreased the most in both oral mastication and relative effects, with both values being 41%, along with an absolute difference of 1.14. Conversely, pollock fish decreased the least in maximum force, with values of 13.3% for oral mastication effect and 13.33% for relative effect, and an absolute difference of 0.18. Pumpkin, noodles, cauliflower, and broccoli increased maximum force, with values ranging from 21% to 63% for oral mastication effect, 0.28 to 1.42 for absolute differences, and 27.45% to 59% for relative effect. Cohesiveness was reduced during oral mastication for the hake fish and zucchini omelet by 2.78% and 19%, with absolute differences of 0.02 and 0.13 and relative effects of 2.78% and 19.70%, respectively. Conversely, oral mastication effects on cohesiveness for broccoli and stewed turkey increased by 2% and 20%, along with respective absolute differences and relative effects. Finally, adhesiveness was increased the most for cauliflower (80%) and decreased the most for hake fish (96%), with similar trends observed in absolute differences and relative effects.

#### 3.1.4. Mastication Process Behavior for the Different Dishes

**Figure 5:**
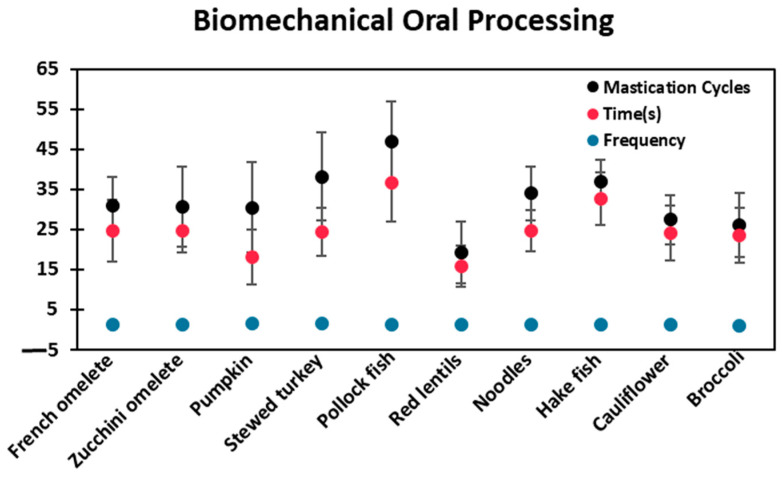
Mean values of the biomechanical mastication parameters: mastication, the time needed for the bolus to be ready, and the frequency of mastication for different dishes. Please note that the masticatory frequency remained quite constant among the dishes.

The results of the biomechanical mastication parameters (mastication cycles, time, and mastication frequency) are presented in Table 7 and Figure 5 for all the fork-mashable dishes analyzed. The results revealed considerable heterogeneity in mastication cycles (MC), with pollock fish requiring the highest number of cycles at 46.87 ± 10.07 MCs, while red lentils displayed a significantly (*p* < 0.05) lower count, i.e., 19.13 ± 7.73 MCs (59.18% variability). The time needed for bolus readiness also exhibited substantial variability, with pollock fish demanding the longest preparation time, at 36.73 s, and red lentils presenting a notably shorter time, at 15.80 ± 5.05 s (56.98% variability) (*p* < 0.05). In contrast, masticatory frequency shows a more constant pattern among dishes: pumpkin, 1.68 ± 0.43 (MC/T); broccoli, 1.11 ± 0.27 (MC/T) (33.92% variability).

## 4. Discussion

The aim of this study was to evaluate the textural qualities of ten fork-mashable dishes designed according to the Triple Adaptation of the Mediterranean Diet for Older People with Oropharyngeal Dysphagia [21] by using a TPA experimental protocol. The main result of this study is that, despite all dishes matching the same qualitative textural descriptors for dysphagia diets (BDA level E or IDDSI level 6), they present a significantly large variation in their textural properties (maximum force, cohesiveness, and adhesiveness) when measured in a quantitative manner using SI units. These large differences in textural properties greatly contribute to the heterogeneity of the oral-processing biomechanics needed to achieve the ready-to-swallow bolus in healthy adults. Taken together, our results argue against the use of subjective descriptors (BDA or IDDSI) for texture-modified food designed for patients with mastication and/or swallowing impairments, as they can include foods with very heterogeneous textural properties and different oral-processing needs under the same descriptor level.

In this study, we investigated the textural and biomechanical properties of fork-mashable texture-modified dishes used to manage patients with oropharyngeal dysphagia. This is a common problem within the patients with dysphagia, as was already explained in a recent study published by our group, showing that, out of the patients who are admitted to the hospital, 48% needed fluid adaptation with a xanthan gum-based thickener (89.4% at 250 mPa·s; 10.6% at 800 mPa·s) and 93.2% needed a texture-modified diet (TMD) (74.4%, fork-mashable; 25.6%, pureed)) [26]. Pre-mastication textural measurements showed that the maximum force varied by 76.19%, cohesiveness by 40.22%, and adhesiveness by an enormous 98.94%. This variability, even when sharing the same qualitative descriptor from organizations like the British Dietetic Association (BDA) or the International Dysphagia Diet Standardization Initiative (IDDSI), indicates potential imprecision in food preparation for patients with OD based solely on these descriptors. Factors such as oral processing, saliva, shear forces, temperature, and food moisture content can cause substantial variations in maximum force (hardness), cohesiveness, and adhesiveness in health and disease [27].

Careful consideration of these textural properties is needed in the preparation of TMDs for individuals with dysphagia to ensure safe and effective consumption, quality control, and reproducibility. In a previous study, it was found that TPs are commercialized using similar qualitative thickness descriptors on their labels, resulting in completely different viscosities when measured in SI units (mPa·s). Similarly, different qualitative descriptors are used for the same objective viscosity values in mPa·s [17]. SI units should be adopted for textural characterization instead of qualitative ones to ensure more consistency while preparing dishes used in clinical and research applications and product development. An article published by Matsuo in 2023 concentrated on the importance of bolus texture and how understanding the role of mastication in modifying food texture can help in developing dietary interventions for individuals with mastication and/or swallowing difficulties [28]. Our study advocates a shift towards quantitative measurement using SI units to accurately assess textural properties, ensuring the safe and effective development and quality control of TMDs developed by the industry or our healthcare centers and catering. This aligns with the call for standardized terminology and definitions in managing dysphagia through texture-modified foods and thickened fluids. Further clinical studies should also link these textural properties measured in SI units with their therapeutic effect on the safety and efficacy of swallowing and the mastication capacity to further guarantee research reproducibility and objective quality control of these diets. 

The Test of Masticating and Swallowing Solids (TOMASS) is an internationally known test that is used as a swallowing and oral preparation tool for solids food [16]. Initial studies were performed on healthy volunteers and used four quantitative parameters: discrete bites, masticatory cycles, swallows, and time to ingest a single cracker [16]. A preliminary psychometric analysis of the TOMASS in a clinical sample of outpatients with dysphagia suggested that it was a reliable and valid tool (specifically related to the number of swallows per cracker). The TOMASS was then proved to be so in patients with dysphagia and to distinguish between patients with dysphagia and healthy adults [15]. We adapted the TOMASS to evaluate oral preparatory and oral phases of solid foods and texture-modified foods by using EMG, accelerometry, and video recording to improve the accuracy of the assessment of the mastication function [29]. In that initial study, we determined the textural characteristics in SI units for five regular food products and the biomechanics of oral mastication processing needed to achieve the RSB for each product in 12 healthy young volunteers. What we found in that study was that texture was homogenized in the RSB independently of the initial texture of the product, especially for hardness, and that oral processing to achieve RSB was adapted to the textural characteristics of foods by modifying the MC and time but maintaining maximum force [29]. In the present study, we found that the biomechanical masticatory process was very heterogeneous, with MCs ranging between 19 and 46 for the different TMDs, with a variation of 59.18%, while the time needed for the formation of RSB of the same amount of texturized food was between 15 and 36 s (56.98% variability). In contrast, the masticatory frequency remained relatively consistent, ranging from 1.11 to 1.65 MC/T, with a variation of 33.92%, in line with our initial studies using regular food. With regular food, the MC ranged between 8 and 25, with a variation of 68%; the time was 4–15 s, and the frequency was between 1.75 and 2.13 MC/T. The wide range of the texturized food of this study compared to the regular food in regard to the mastication cycles and time might be attributed to the dish composition geometry and weight. These discrepancies highlight the intricate relationship between food texture and mastication behavior. The differences in mastication cycles and bolus preparation time among dishes can be attributed to factors such as the huge differences in food texture in the same BDA/IDDSI descriptors, moisture content, and structural integrity. In addition, the relationships between the food’s physical properties and oral-processing behavior and mastication are yet to be known [30]. 

RSB is a mixture of food and saliva that is formed in the mouth during the process of chewing and before being swallowed. RSB plays a major role in safe swallowing, as it can result in aspiration if boluses are not well prepared during oral processing [3]. Variability in textural properties among RSB formed after mastication indicates the impact of dish texture modification on bolus properties. In our study, RSB showed a maximum force ranging from 0.53 N to 1.59 N, with a variation of 66.66%. Cohesiveness ranged between 0.49 and 0.73, with a variation of 32.37%. Adhesiveness varied from 0.01 to 1.15 N·s, displaying a high variability of 99%. When compared with pre-mastication values, oral processing resulted in reductions in the maximum force, ranging between 13 and 41% in six dishes, but also increments between 21 and 63% were observed in four dishes. Cohesiveness exhibited reductions of between 2 and 19% in five dishes and increments between 2 and 20% in another five. Adhesiveness increased between 16 and 90% for eight dishes and decreased between 58 and 96% for the remaining two. Other research has focused on the importance of understanding the influence of oral breakdown of food on dynamic texture perception, particularly in the context of forming a safe-to-swallow bolus [31]. The granularity of the bolus before swallowing is a critical aspect that needs to reach a predetermined state through individualized mastication strategies [32]. In addition, the relationship between mastication, salivation, and food bolus formation is important for food consumption and highlights the intricate processes involved in oral processing [33]. Comparing mastication work percentages between the pre- and post-oral mastication, we see that the increase in maximum force after mastication using TPA in several dishes could be linked to the initial dish states. Before mastication, soft food particles with air gaps were present in the acrylic recipient of the texture analyzer. Mastication led to particle mixing, attachment, and reduced air gaps, which led to higher compression resistance and increased maximum force [34]. The resulting differences in the textural results of dishes and the bolus could put the patients at risk. However, this effect was observed only in dishes with separated small particles, while other dishes showed a decrease in the maximum force after the mastication [35]. Mastication work led to an increase in adhesiveness due to mixing with saliva, and the cohesiveness varied depending on the dish [36].

The main limitation of our study is that, before mastication, not all dishes had the same geometry, and, although all were 30 g, this might have affected texture measurements. Geometry mainly affects the pre-bolus, as after mastication, they are all of a cohesive bolus puree type. In future studies, it would be interesting to analyze more dishes and larger sample sizes (participants) and incorporate patients instead of volunteers. Future research should use TPA protocols with textural measurements to measure in SI units both the pre- and post-mastication of different dishes in order to understand how the oral behavior of the patients affects and converts the food from the fork-mashable to swallowable bolus. Both the TOMASS and V-VST should be used to check the force, time, and frequency needed to prepare those boluses and V-VST if they are safe to swallow by the patients in a clinical trial and link them to the textural properties obtained from the TPA until they have a strong correlation between bolus preparation and safety of swallowing with their textural properties. This research pathway is similar to that performed on alimentary fluids and thickened fluids in patients with swallowing disorders [37]. 

## 5. Conclusions

In conclusion, this study found significant variations in the textural properties (maximum force, cohesiveness, and adhesiveness) of dishes classified under the same qualitative descriptor level (BDA level E/IDDSI level 6), indicating a risk for patients consuming them. Maximum force values ranged from 0.65 to 2.73 N for pre-mastication and from 0.70 to 2.24 N post-mastication. Additionally, the number of masticatory cycles and the time required to achieve an RSB differed significantly among dishes, with mastication cycles ranging from 46.87 to 19.13 and time from 36.73 to 15.80 s, while masticatory frequency remained relatively constant (1.68–1.11 MC/T). This shows an urgent need for standardized evaluation protocols using objective measurements in SI units to ensure consistency in texture-modified diets. 

## Figures and Tables

**Table 3:** An estimation of the ingredient’s percentage, weight in g per recipe, protein, and number of kilocalories for all the thick purees tested. The Nutilis Clear (xanthan gum-based) thickener is referred to as weight in g.

Puree	Ingredients	Weight/Recipe (g)	Carbohydrates (g)	Proteins (g)	Kcal
French omelet	86.2% eggs in a French omelet, 1.4% water, and 1.1% virgin olive oil	100	0.29	7.17	156.56
Zucchini omelet	44.6% zucchini, 8.9% ratatouille *, 0.2% salt, 1.8% virgin olive oil, 26.7% diced potatoes, and 17.8% water	100	0.39	2.90	103.62
Pumpkin	80.4% diced pumpkin, 16.1% carrot, 0.3% salt, 3.1% virgin olive oil, and 0.99 thickener	100	0.21	1.18	101
Stewed turkey	23.1% sliced turkey breast, 1.3% salt, 5.3% virgin olive oil, 0.5% black pepper, 18.41% water, 8.8% concentrated chicken broth without salt, 19.4% ratatouille *, 13.3% diced potato, 9.1% diced carrot, and 0.99 thickener	100	0.5	13.41	151.15
Pollock fish	35.72% pollock fish, 0.33% salt, 1.56% olive oil, 0.11% black pepper, and 61.29% lemon sauce, and 0.99 thickener	100	-	10.21	113.18
Red lentils	15.6% lentils, 31.3% ratatouille *, 3.9% concentrated chicken broth, 0.1% salt, 2% extra virgin olive oil, 18.4% diced potato, 7.8% diced carrot, 19.6% water, and 0.2% ground cumin, 0.99 thickener.	100	12.57	6.93	134.76
Noodles	18.7% nº 2 noodles, 1.9% olive oil, 0.9% salt, 9.3% ratatouille *, 55% stock, 5.6% peeled shrimp, 1.9% garlic and parsley, 5.6% mussels, and 1% Nutilis Clear thickener	100	13	4.9	141.81
Hake fish	94.2% hake fish, 0.9% salt, 3.8% olive oil, and 0.4% black pepper, and 0.98 thickener	100	-	10.19	113.1
Cauliflower	44.6% cauliflower, 8.9% ratatouille *, 0.2% salt, 1.8% virgin olive oil, 24.7% diced potato, and 17.8% water	100	5.3	2.75	74.48
Broccoli	44.6% broccoli, 8.9% ratatouille *, 0.2% salt, 1.8% virgin olive oil, 24.7% diced potato, and 17.8% water	100	6	2.75	74.48

* Ratatouille is a mixture of vegetables, including water, onions, carrots, celery.

**Table 4:** TPA calculated mean value of maximum force at pre- and post-mastication and the calculated percentages of the oral-processing effect, absolute differences between pre- and post-mastication and the relative effect on the pre-mastication.

Texture-Modified Fork Mashable Diet	Maximum Force (N) Pre-Mastication	Maximum Force (N) Post-Mastication	% Oral Mastication Effect	Absolute Differences (Pre–Post)	Relative Effect on Pre-Mastication, %
	Mean (SD)	Mean (SD)			
French omelet	1.06 abc (0.81)	0.70 ab (0.30)	34	0.36	33.96
Zucchini omelet	2.73 d (1.40)	1.59 de (0.88)	41	1.14	41.78
Pumpkin	1.02 ab (0.45)	1.30 bcd (0.62)	21.5	0.28	27.45
Stewed turkey	1.69 abc (0.68)	1.08 abcd (0.39)	36.1	0.61	36.09
Pollock fish	1.35 bcd (0.75)	1.17 abcd (0.52)	13.3	0.18	13.33
Red lentils	1.06 abc (0.41)	0.88 abc (0.29)	17	0.72	67.92
Noodles	0.83 ab (0.45)	1.36 abcd (0.84)	39	0.53	63.86
Hake fish	1.11 abc (0.54)	0.53 a (0.18)	52.8	0.58	52.25
Cauliflower	0.65 ab (0.17)	1.54 cde (0.58)	57.8	0.89	41
Broccoli	0.82 a (0.09)	2.24 e (1.13)	63.4	1.42	59

Different letters in the same column indicate significant differences (*p* < 0.05) among the maximum force of the different dishes according to Tukey’s test.

**Table 5:** TPA-calculated mean value of cohesiveness at pre- and post-mastication and the calculated percentages of the oral-processing effect, absolute differences between pre- and post-mastication, and relative effect on the pre-mastication.

Texture-Modified Fork Mashable Diet	Cohesiveness Pre-Mastication	Cohesiveness Post-Mastication	% Oral Mastication Effect	Absolute Differences (Pre–Post)	Relative Effect on Pre-Mastication, %
	Mean (SD)	Mean (SD)			
French omelet	0.87 e (0.03)	0.73 d (0.13)	16.09	0.14	16.09
Zucchini omelet	0.66 cd (0.05)	0.53 a (0.05)	19.70	0.13	19.70
Pumpkin	0.52 b (0.05)	0.54 ab (0.05)	3.7	0.02	3.85
Stewed turkey	0.50 b (0.13)	0.63 bc (0.09)	20.6	0.13	26
Pollock fish	0.76 de (0.05)	0.72 d (0.05)	5.26	0.04	5.26
Red lentils	0.57 bc (0.14)	0.69 cd (0.06)	17.4	0.12	11.32
Noodles	0.53 bc (0.09)	0.49 a (0.06)	7.55	0.04	4.82
Hake fish	0.72 d (0.05)	0.70 cd (0.06)	2.78	0.02	2.78
Cauliflower	0.49 b (0.04)	0.50 a (0.04)	2	0.01	2.04
Broccoli	0.58 a (0.04)	0.52 a (0.06)	10.34	0.06	10.34

Different letters in the same column indicate significant differences (*p* < 0.05) among the cohesiveness of the different dishes according to Tukey’s test.

**Table 6:** TPA-calculated mean value of adhesiveness at pre- and post-mastication and the calculated percentages of the oral-processing effect, absolute differences between pre- and post-mastication, and relative effect on the pre-mastication.

Texture-Modified Fork Mashable Diet	Adhesiveness (N·s) Pre-Mastication	Adhesiveness (N·s) Post-Mastication	% Oral Mastication Effect	Absolute Differences (Pre–Post)	Relative Effect on Pre-Mastication, %
	Mean (SD)	Mean (SD)			
French omelet	0.12 a (0.45)	0.05 a (0.07)	58	0.07	58.33
Zucchini omelet	0.04 a (0.02)	0.20 b (0.23)	80	0.16	13.04
Pumpkin	0.15 ab (0.10)	0.63 a (0.26)	76.2	0.48	10.43
Stewed turkey	0.66 bc (0.74)	1.15 a (0.39)	42.6	0.49	74.24
Pollock fish	0.01 a (0.01)	0.03 a (0.03)	66.7	0.02	6.52
Red lentils	0.95 c (0.47)	1.14 a (0.27)	16.7	0.19	20
Noodles	0.06 a (0.07)	0.30 a (0.40)	80	0.24	13.04
Hake fish	0.25 ab (0.99)	0.01 a (0.01)	96	0.24	96
Cauliflower	0.02 a (0.01)	0.22 a (0.14)	90.9	0.2	32.60
Broccoli	0.04 a (0.02)	0.34 a (0.24)	88.2	0.3	24.45

Different letters in the same column indicate significant differences (*p* < 0.05) among the adhesiveness according to Tukey’s test.

**Table 7:** Masticatory work calculated mean value of mastication cycles, time, and mastication frequency.

Texture-Modified Fork Mashable Diet	Mastication Cycles	Time (s)	Mastication Frequency (MC/T)
	Mean (SD)	Mean (SD)	Mean (SD)
French omelet	30.92 abc (8.16)	24.63 ab (8.89)	1.26 ab (0.15)
Zucchini omelet	30.60 abc (9.91)	24.67 ab (5.41)	1.24 ab (0.21)
Pumpkin	30.50 abc (12.85)	18.18 a (6.82)	1.68 a (0.43)
Stewed turkey	38.20 bc (11.10)	24.27 ab (5.99)	1.57 b (0.14)
Pollock fish	46.87 c (10.07)	36.73 b (9.83)	1.28 ab (0.22)
Red lentils	19.13 a (7.73)	15.80 a (5.05)	1.21 ab (0.12)
Noodles	33.97 abc (6.69)	24.60 ab (5.13)	1.38 ab (0.17)
Hake fish	37.00 abc (5.20)	32.60 b (6.47)	1.13 ab (0.16)
Cauliflower	27.40 ab (6.05)	24.13 ab (6.76)	1.14 ab (0.27)
Broccoli	26.13 ab (8.03)	23.47 ab (6.78)	1.11 ab (0.27)

Different letters in the same column indicate significant differences (*p* < 0.05) according to Tukey’s test.

## Data Availability

The original contributions presented in the study are included in the article/Supplementary Materials, further inquiries can be directed to the corresponding author.

## Supplementary Materials

The following supporting information can be downloaded at https://www.mdpi.com/article/10.3390/foods13121807/s1, Figure S1: Selected Mediterranean thick purees for this study.
