# Supplementary material for: Exploring Texture and Biomechanics of Food Oral Processing in Fork-Mashable Dishes for Patients with Mastication or Swallowing Impairments
Source: Foods. 2024 Jun 8;13(12):1807. doi: 10.3390/foods13121807 (PMC11202756; doi:10.3390/foods13121807)
Supplement: Supplementary file 1 [file foods-13-01807-s001.zip › foods-3002427-supplementary.pdf]

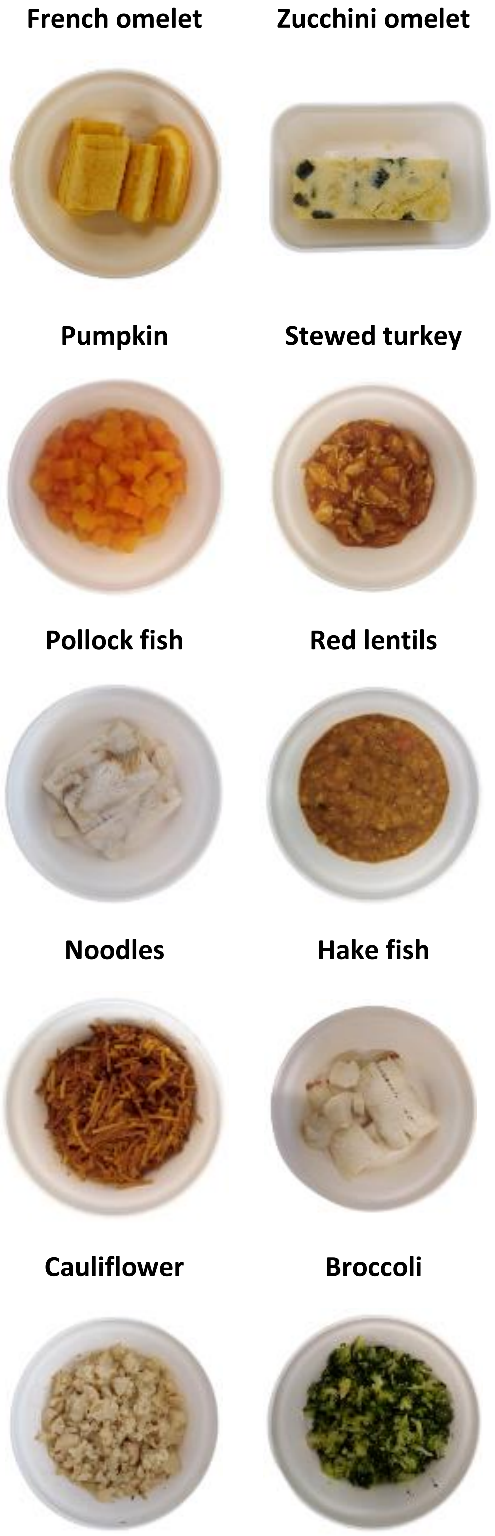

Figure S1: Selected Mediterranean Fork-Mashable dishes for the study.

Cooking Method;

The cooking methods for these fork-mashable dishes are as follows. The French omelet was steamed at 100°C for 12 minutes using wet heat. The zucchini omelet involved cooking the potatoes in the oven at 180°C with 30% humidity for 30 minutes. Separately, zucchini was steamed in the oven at 100°C for 20 minutes. After cooking, the zucchini and potatoes were mixed with pasteurized liquid egg and cooked together in the oven at 115°C with 99% humidity for 35 minutes, using a combination of dry and wet heat. Pumpkin was steamed in the oven at 100°C for 25 minutes using wet heat. For the stewed turkey, turkey breast was trimmed of nerves and fat, cut into small pieces, and browned in a pot over direct heat. Once browned, it was cooked with a base of vegetable broth, diced carrots, and water for approximately 1 hour and 20 minutes over medium heat until the meat was tender and could be shredded, utilizing a combination of dry and wet heat. Pollock fish was steamed at 100°C for 12 minutes using wet heat. Red lentils were cooked in a pot with vegetable broth, diced carrots, diced potatoes, and a base of vegetable ratatouille for 30 minutes over medium heat using wet heat. Noodles were toasted in the oven. Shrimp were sautéed in a pot with a garlic/parsley mixture until browned, then mixed with a base of noodles and the toasted noodles. Fish broth was added, and the mixture was cooked for 2 minutes over high heat. The heat was then turned off to allow the noodles to absorb the broth, using a combination of dry and wet heat. Hake fish was steamed in the oven at 100°C for 12 minutes using wet heat. Broccoli was steamed in the oven at 100°C for 30 minutes using wet heat, and cauliflower was steamed in the oven at 100°C for approximately 25-30 minutes using wet heat. Finally about 1 gram of the thickener was added to noodles, pumpkin, stewed turkey, pollock fish, hake fish and red lentils.
